# Supplementary material for: Revisiting Minamata disease through computational phenotypic similarity analysis
Source: PLoS One. 2026 Feb 26;21(2):e0342655. doi: 10.1371/journal.pone.0342655 (PMC12944806; doi:10.1371/journal.pone.0342655)
Supplement: S1 Table — Formatted table of symptoms of patients from the Minamata Area diagnosed with Minamata disease as found in [13], with the associated frequency and the most closely related HPO terms. Notice that the frequency of “Mental retardation" was inconsistently annotated in [13]. To resolve the contradiction, we selcted the smaller reported frequency (0.37%). (PDF) [file pone.0342655.s001.pdf]

Symptom	HPO terms	Frequency
Mental retardation	HP:0001249 Intellectual disability	0.003717
Schizophrenia	HP:0100753 Schizophrenia	0.003717
Neurotic condition	HP:0000739 Anxiety	0.081784
Schizophrenic type condition	HP:0000725 Psychotic episodes	0.007435
Manic-depressive condition	HP:0007302 Bipolar affective disorder	0.059480
Sensory Disorder	HP:0003474 Sensory impairment	0.970000
High Blood Pressure	HP:0032263 Increased blood pressure	0.498000
Epilepsy	HP:0002133 Status epilepticus	0.089000
Loss of Vegetable Function	HP:0000741 Apathy	0.613000
Loss of Smell and Taste	HP:0000458 Anosmia	0.338000
	HP:0000223 Abnormality of taste sensation	
Hearing Loss	HP:0000365 Hearing impairment	0.840000
Narrow Vision	HP:0001133 Constriction of peripheral visual field	0.595000
Parkinsonism	HP:0001300 Parkinsonism	0.032000
Jerky Movement	HP:0001257 Spasticity	0.245000
Excess Movement	HP:0100022 Abnormality of movement	0.952000
Poor Reflex	HP:0002600 Hyporeflexia of lower limbs	0.182000
Abnormal Inherent Reflex	HP:0007034 Generalized hyperreflexia	0.822000
Loss of Muscle Tension	HP:0001252 Hypotonia	0.145000
Muscle Tension	HP:0003552 Muscle stiffness	0.632000
Loss of Strength	HP:0001324 Muscle weakness	0.673000
Loss of Balance	HP:0002172 Postural instability	0.937000
Speech Problem	HP:0002167 Abnormal speech pattern	0.625000
