# Supplementary material for: Revisiting Minamata disease through computational phenotypic similarity analysis
Source: PLoS One. 2026 Feb 26;21(2):e0342655. doi: 10.1371/journal.pone.0342655 (PMC12944806; doi:10.1371/journal.pone.0342655)
Supplement: S2 Table — Rankings of diseases most similar to Minamata disease using five approaches (Jaccard Index, TF-IDF, TF-IDF with query expansion, Resnik and GraphIC) with the associated scores. (PDF) [file pone.0342655.s002.pdf]

Similarity	Disease name	Score
**Jaccard**	X-linked spasticity-intellectual disability-epilepsy syndrome	0.153
	cyanide-induced parkinsonism	0.131
	PRKAR1B-related neurodegenerative dementia with intermediate filaments	0.121
	early-onset parkinsonism-intellectual disability syndrome	0.121
	encephalopathy, recurrent, of childhood	0.121
	progressive supranuclear palsy-parkinsonism syndrome	0.113
	peroxisome biogenesis disorder 9B	0.111
	isolated cerebellar hypoplasia/agenesis	0.111
	intellectual disability, X-linked 63	0.111
	beta-mannosidosis	0.108
**TF-IDF**	psychogenic movement disorders	0.376
	choreatic disease	0.291
	cyanide-induced parkinsonism	0.266
	HSD10 disease, atypical type	0.264
	encephalopathy, recurrent, of childhood	0.255
	lateral sclerosis	0.250
	Spasmus nutans	0.226
	early-onset parkinsonism-intellectual disability syndrome	0.220
	neuronal intranuclear inclusion disease	0.220
	spinocerebellar ataxia type 18	0.212
**TF-IDF with QE**	psychogenic movement disorders	0.160
	spinocerebellar ataxia type 18	0.157
	cyanide-induced parkinsonism	0.155
	progressive supranuclear palsy-corticobasal syndrome	0.148
	torsion dystonia 13	0.147
	corticobasal syndrome	0.144
	atypical juvenile parkinsonism	0.139
	Kufor-Rakeb syndrome	0.138
	lateral sclerosis	0.137
	dyskinesia with orofacial involvement, autosomal dominant	0.135
**Resnik**	Kufor-Rakeb syndrome	2.42
	burning mouth syndrome	2.32
	progressive supranuclear palsy-parkinsonism syndrome	2.22
	autosomal recessive early-onset Parkinson disease 6	2.19
	parkinson disease 25, autosomal recessive early-onset	2.16
	cyanide-induced parkinsonism	2.14
	autosomal recessive early-onset Parkinson disease 7	2.13
	PRKAR1B-related neurodegenerative dementia with intermediate filaments	2.13
	late-onset Parkinson disease	2.08
	young-onset Parkinson disease	2.07
**GraphIC**	Susac syndrome	0.632
	Huntington disease-like 2	0.614
	encephalopathy, recurrent, of childhood	0.613
	juvenile onset Parkinson disease 19A	0.598
	Kufor-Rakeb syndrome	0.597
	guanidinoacetate methyltransferase deficiency	0.591
	Tay-Sachs disease AB variant	0.585
	basal ganglia calcification, idiopathic, 1	0.583
	young-onset Parkinson disease	0.575
	encephalopathy due to GLUT1 deficiency	0.575
