# Supplementary material for: Revisiting Minamata disease through computational phenotypic similarity analysis
Source: PLoS One. 2026 Feb 26;21(2):e0342655. doi: 10.1371/journal.pone.0342655 (PMC12944806; doi:10.1371/journal.pone.0342655)
Supplement: Attachment — Submitted filename: RebuttalPlosOne_2025.pdf [file pone.0342655.s006.pdf]

We thank the Editor and reviewers for their positive comments. Following a point by point rebuttal.

1. Editor: Please ensure that your manuscript meets PLOS ONE's style requirements, including those for file naming. The PLOS ONE style templates can be found at https://journals.plos.org/plosone/s/file?id=wjVg/PLOSOne_formatting_sample_main_body.pdf .

   Answer: We have followed the requirements.

2. Editor:Thank you for stating the following financial disclosure: [CAMLP and EC acknowledge funding from FAIR - Future Artificial Intelligence Research: Adaptive AI methods for Digital Health" grant number PNRR_BAC24GVALE_01 PE_0000013, CUP D53C2200238000]. Please state what role the funders took in the study. If the funders had no role, please state: "The funders had no role in study design, data collection and analysis, decision to publish, or preparation of the manuscript".

   Answer: We added the sentence to the financial disclosure: "The funders had no role in study design, data collection and analysis, decision to publish, or preparation of the manuscript"

   Editor: Please note that PLOS One has specific guidelines on code sharing for submissions in which author-generated code underpins the findings in the manuscript. In these cases, we expect all author-generated code to be made available without restrictions upon publication of the work. Please review our guidelines at https://journals.plos.org/plosone/s/materials-and-software-sharing#loc-sharing-code and ensure that your code is shared in a way that follows best practice and facilitates reproducibility and reuse.

   Answer: the code is publicly available in GitHub.

3. Editor: When completing the data availability statement of the submission form, you indicated that you will make your data available on acceptance. We strongly recommend all authors decide on a data sharing plan before acceptance, as the process can be lengthy and hold up publication timelines. Please note that, though access restrictions are acceptable now, your entire data will need to be made freely accessible if your manuscript is accepted for publication. This policy applies to all data except where public deposition would breach compliance with the protocol approved by your research ethics board. If you are unable to adhere to our open data policy, please kindly revise your statement to explain your reasoning and we will seek the editor's input on an exemption. Please be assured that, once you have provided your new statement, the assessment of your exemption will not hold up the peer review process

   Answer: all the relevant data is available as supplementary information files.

4. Editor: If the reviewer comments include a recommendation to cite specific previously published works, please review and evaluate these publications to determine whether they are relevant and should be cited. There is no requirement to cite these works unless the editor has indicated otherwise.

Answer: We didn’t receive any review comments

5. Editor:Please review your reference list to ensure that it is complete and correct. If you have cited papers that have been retracted, please include the rationale for doing so in the manuscript text, or remove these references and replace them with relevant current references. Any changes to the reference list should be mentioned in the rebuttal letter that accompanies your revised manuscript. If you need to cite a retracted article, indicate the article’s retracted status in the References list and also include a citation and full reference for the retraction notice.

   Answer: The reference list does not contain any retracted papers.

6. Additional Editor Comments: Please resubmit with higher quality images to facilitate the review process for our referees. It is currently not possible to read some of the figures

   Answer: We provide now higher quality figures.

7. Reviewers' comments:

   Answer: We didn’t receive any reviewer’s comments.
