# Supplementary material for: Revisiting Minamata disease through computational phenotypic similarity analysis
Source: PLoS One. 2026 Feb 26;21(2):e0342655. doi: 10.1371/journal.pone.0342655 (PMC12944806; doi:10.1371/journal.pone.0342655)
Supplement: Attachment — Submitted filename: Response to Reviewer.pdf [file pone.0342655.s007.pdf]

Response to Reviewer #1

Concern 1: *There are many studies that use the similarity of HPO to evaluate disease distance. for example, RDmap: A Map for Exploring Rare Diseases. Orphanet Journal of Rare Diseases. 2021, 16(101). It calculates distances for thousands of rare diseases and visualizes them. It seems that this tool can be used to complete the search for diseases with similar phenotypes to a certain disease in this study.*

Answer: We thank the reviewer for pointing us to RDmap and related work. We now explicitly cite RDmap in the Introduction and discuss how our study relates to this line of research. While tools such as RDmap are invaluable for the interactive exploration of rare diseases already present in ontological databases, our work is unique in its ability to integrate and leverage unstructured historical clinical data. Our methodology can be extended to other environmental or historical diseases not yet formalized in major databases, providing a replicable approach for extracting value from legacy epidemiological data. In particular, our work focuses on Minamata disease as a case study of a disorder that is **not** natively represented in HPO or OMIM and whose clinical description originates from historical epidemiological surveys.

We have added a paragraph in the introduction clarifying this distinction and framing our study as complementary to RDmap—relying on similar ontological infrastructure but addressing a different problem setting and data type. See **page 2 lines 39-42**.

Concern 2: *The search for diseases with similar phenotypes is quite straightforward for rare diseases. The Minamata disease targeted in this study has nothing special about it. Clearly, if another disease were to be found, a list of similar diseases would also be obtained. These lists may imply some disease mechanisms, but all of these need to be verified through subsequent research. This research clearly lacks such a conclusion.*

Answer: We agree that, from a technical standpoint, once diseases are represented in a standardized ontology such as HPO, searching for phenotypically similar disorders is conceptually straightforward and can be applied to any index disease. The added value of our work lies in the practical demonstration of how historical epidemiological data can be integrated into the context of modern ontologies, enabling systematic comparisons and the generation of new hypotheses. Specifically, the application to Minamata disease shows how it is possible to explore the position of an environmental disease within the landscape of neurological disorders, even in the absence of genetic or molecular data. This approach may be useful for other environmental or multifactorial diseases, which are often overlooked by genetic databases.

In the revised version of the manuscript, we have better clarified in the Introduction and Discussion that:

- Our goal is **not** to introduce a novel similarity algorithm, but to demonstrate a reproducible workflow for translating historical clinical descriptions (here, Minamata disease) into ontological form and contextualizing them within the modern landscape of >12,000 HPO-annotated diseases.

- Minamata disease serves as an important case study because it represents a prototypical environmental neurotoxic disorder with a well-documented clinical picture, yet it is absent from modern disease ontologies and databases.

- The resulting similarity rankings are intended as **hypothesis-generating**, not as definitive mechanistic proofs. We now state explicitly that any mechanistic inferences suggested by phenotypic similarity must be tested in future experimental and epidemiological studies.

We discuss the point above at end of the Introduction **page 2 lines 45-52**.

Furthermore, to address the concern about the lack of clear conclusions, we have added a short **Conclusions** section (**page 10**) that:

- Summarizes the main consistent findings, including the convergence of multiple metrics on movement disorders and neurodegenerative or neurotoxic syndromes (notably cyanide-induced parkinsonism).
- Emphasizes that these patterns support the plausibility of shared affected neuroanatomical systems (e.g., cerebellar and extrapyramidal involvement) without asserting a shared molecular mechanism.
- Clearly delineates the limitations of phenotypic similarity as a standalone tool and the need for independent validation.

Concern 3: *In addition, the core focus of the article is somewhat confusing. In the method section, a large number of similarity calculation methods are introduced, with the focus on the technical aspect. However, the results section does not pay much attention to these methods but instead obtains a comprehensive ranking list, which mainly presents various possible hypotheses. Essentially, this research lacks a conclusion.*

Answer: We appreciate this comment and have revised parts of the manuscript to clarify the core focus and improve the alignment between Methods and Results. In the revised Introduction, we now explicitly state that the main objective is to derive *robust* disease rankings by combining multiple complementary similarity metrics and then interpret the resulting consensus in terms of disease categories and neurotoxic vs genetic etiologies (**page 2**). This makes clear why we devoted less space to different metrics, but ultimately centered the results on consensus patterns. In the revised Results section (**pages 6-7 lines 225-245**), we now make more explicit reference to how the individual metrics behave and where they agree or differ, rather than immediately focusing only on the aggregated ranking. As mentioned above, we added a more explicit Conclusions section that synthesizes the methodological and biological insights and clarifies the scope and limits of the work.

***Reviewer #2**: The manuscript presents a clear and well-structured computational analysis that maps historical Minamata disease symptoms to HPO terms and compares them against >12,000 diseases using multiple similarity metrics. The scope and framing align with PLOS ONE's focus on technical rigor and ethical standards rather than perceived impact.*
The methods are described in commendable detail, including ontology mapping, frequency harmonization, and rank aggregation.

***Concern 1**: Two preprocessing choices would benefit from small additions to improve reproducibility:*

1. Provide a brief sensitivity analysis showing the effect of assigning frequency 0.5 to symptoms lacking data (currently justified as an uninformative prior)

Answer: We now report a sensitivity analysis using two extreme values that correspond to HPO frequency classes: 0.01 ("very rare", HP:0040284) and 1.0 ("obligate", HP:0040280). For each choice, we recomputed all similarity rankings and the main figures. Conclusions are qualitatively unchanged: (i) the sharp score drop after ~100 diseases persists; (ii) cross-method rank concordance remains high and the pattern is the same of f=0.5; (iii) the same diseases and disease families remain at the top, although with slightly different rankings. We summarize these results in Fig. S1 and S2. We also add a short note in *Results* stating that our findings are robust even to these extreme bounds on unknown frequencies and a brief comment in *Methods* stating that a sensitivity analysis has been performed (**page 3 lines 83-85**).

2. Justify the query-expansion decay factor α = 0.5 with either literature precedent or a data-driven comparison (e.g., α ∈{0.3, 0.5, 0.7}) on rank stability.

Answer: The factor *α* acts as a trade-off between specificity (low *α*) and inclusion of neighbouring phenotypes (high *α*). We performed a comparison over α ∈ {0 (no QE), 0.1, 0.3, 0.5, 0.7, 0.9, 1.0}. The Kendall-τ heatmap (included as Fig. S3) shows a stability plateau for α≈0.3–0.7. Rankings at α=0.5 are highly concordant with α=0.3 (τ≈0.96) and α=0.7 (τ≈0.94), and remain well aligned with the non-expanded TF-IDF baseline (τ≈0.77) which corresponds to α=0. Concordance drops as α→1.0, which corresponds to the case where all neighbours are assigned the same frequency of the original phenotypes. We therefore keep α = 0.5 as a neutral mid-range value within this plateau, representing a fair trade-off between preserving specificity and capturing nearby ontology neighbours. We now justify this choice in the *Methods* section (**page 5 lines 183-186**).
The results are compelling and consistently converge on movement and neurodegenerative disorders, with cyanide-induced parkinsonism repeatedly appearing near the top of multiple rankings. Figure 2b's weighted Kendall τ visualization effectively communicates agreement among metrics, and Figure 3's consensus ranking is appropriate.

Concern 2*: To strengthen interpretation while remaining within PLOS ONE's rigor criteria, consider adding a short paragraph that distinguishes likely mechanistic overlap (for example, mitochondrial dysfunction, oxidative stress) from coincidental phenotypic similarity, and that clarifies which top matches plausibly relate to environmental neurotoxicology versus genetic etiologies. This will better align claims to the supporting data and help readers understand the limits of phenotypic similarity alone.*

Answer: We added a brief interpretive paragraph to *Discussion* distinguishing:
(i) toxin-linked entities (e.g., cyanide-induced parkinsonism; mercury-related encephalopathy), which are compatible with environmental neurotoxicology mechanisms, and
(ii) genetic neurodegenerative disorders (e.g., PRKAR1B-related dementia…), where proximity likely coincidental phenotypic similarity rather than shared etiology.

See **pages 9-10 lines 359-374**.

Concern 3:
Finally, a few presentation details will improve clarity without altering conclusions. In the "Minamata Disease symptoms dataset" please cite the specific tables used to resolve the discrepancy for "Mental Retardation" and, if possible, add a short note in S1 Table that flags this correction. In the figures, ensure axis labels are fully legible in the final PDF.

Answer*:* In *Minamata Disease symptoms dataset*, we now cite the exact tables used to resolve the "Mental Retardation" frequency discrepancy and add a note in S1 Table flagging the corrected frequency. **See page 4 lines 111-115.**
